# Supplementary material for: THINGS-data, a multimodal collection of large-scale datasets for investigating object representations in human brain and behavior
Source: eLife. 2023 Feb 27;12:e82580. doi: 10.7554/eLife.82580 (PMC10038662; doi:10.7554/eLife.82580)
Supplement: Supplementary file 1. [file elife-82580-supp1.docx]

## Supplementary File 1: Magnetic resonance imaging acquisition parameters

|  | Main task | Resting state | pRF | Category localizer | T1w | T2w | T2* | TOF | Field map |
| --- | --- | --- | --- | --- | --- | --- | --- | --- | --- |
| Sequence type | GE-EPI | | | | MPRAGE | SPACE | 3D-EPI | Multi-slab | Gradient echo |
| Resolution [mm] | 2 (iso) | | | | 0.8 (iso) | 0.8 (iso) | 0.7 (iso) | 0.3 × 0.3 × 0.5 | 3 (iso) |
| # Volumes | 284 | 240 | 180 | 308 | 1 | | | | |
| FOV [mm] | 192 × 192 | | | | 256 × 40 | | 269 × 218 | 230 × 209 | 192 × 192 |
| Matrix size | 96 × 96 | | | | 320 × 300 | | 384 × 312 | 768 × 696 | 64 × 64 |
| TR [s] | 1.5 | | | | 2.4 | 3.2 | 0.064 | 0.021 | 0.52 |
| TE [ms] | 33 | | | | 2.24 | 564 | 35 | 3.43 | 520 |
| Flip angle | 75° | | | | 8° | 120° | 10° | 18° | 60° |
| Slice orientation | Axial | | | | Sagittal | | | Axial | |
| Phase encoding direction | P >> A | A >> P | P >> A | | A >> P | | | R >> L | P >> A |
| Number of slices | 60 | | | | 208 | | 256 | 232 | 49 |
| Slice thickness [mm] | 2 | | | | 0.8 | | 0.65 | 0.5 | 3 |
| Distance factor [%] | 0 | | | | 50 | 0 | 50 | -20 | 0 |
| Order of slice acquisition | Interleaved | | | | | | | Ascending | Interleaved |
| Parallel imaging sequence | Multiband | | | | GRAPPA | | None | GRAPPA | None |
| Acceleration factor | 3 | | | | 2 | | None | 2 | None |
| Bandwidth [Hz/px] | 2,264 | | | | 210 | 744 | 394 | 186 | 300 |
